# A Cross-Sectional Study of Self-Rated Health among Older Adults: Association with Drinking Profiles and Other Determinants of Health

**DOI:** 10.1155/2015/352947

**Published:** 2015-12-30

**Authors:** Pascale Audrey Moriconi, Louise Nadeau

**Affiliations:** Department of Psychology, Université de Montréal, CP 6128, Succursale Centre-Ville, Montréal, QC, Canada H3C 3J7

## Abstract

This study compares the relationship between drinking profiles and self-rated health with and without adjusting for other determinants of health among a sample of older adults from the general population. Respondents were 1,494 men and 2,176 women aged between 55 and 74 from the GENACIS Canadian survey. The dependent variable was self-rated health, an individual's perception of his or her own general health, a measure used as a proxy for health status. The independent variables were drinking profiles (types of drinkers and nondrinkers) as well as other demographic, psychosocial, and health-related variables (control variables). After adjustment for other determinants of health, regression analyses showed that (1) frequent/moderate drinkers were more likely to have a better self-rated health compared with nondrinkers (lifetime abstainers and former drinkers) and (2) self-rated health did not differ significantly between frequent/moderate drinkers and other types of drinkers (frequent/nonmoderate and infrequent drinkers). Our results suggest that drinking is related to a better self-rated health compared with nondrinking regardless of the drinking profile. Drinking and healthy lifestyle guidelines specific to older adults should be studied, discussed, and integrated into public health practices.

## 1. Introduction

Population aging is a worldwide phenomenon, caused mainly by an increase in life expectancy combined with a decrease in fertility rates [1]. Evidence suggests that the number of individuals aged 60 years and older will exceed the number of children by 2047. This major shift has important repercussions on health, health systems, and budgets and has consequently brought global attention to health-related domains.

In alcohol research, the topic of older adults' health has traditionally been controversial. On one hand, older adults are more likely to experience adverse effects of alcohol (due to higher and longer-lasting blood alcohol concentrations caused by normal physiological changes with aging), to suffer from chronic diseases and to use medication that may interact with alcohol [2–4]. On the other hand, older adults are more likely to benefit from moderate levels of alcohol consumption. The protective effect of moderate drinking on all-cause mortality and cardiovascular morbidity and mortality [5–10] has been shown to increase in middle-aged and older adults [11–15]. A J-shaped relationship has been found, where abstainers have a higher mortality risk than moderate drinkers, whereas excessive (heavy) drinkers have a higher mortality risk than both abstainers and moderate drinkers [6, 9, 10, 16]. A meta-analysis showed the lowest risk of coronary heart disease mortality among those who reported 1-2 drinks per day; however, for stroke mortality, the lowest risk occurred among those who reported 1 drink or fewer per day [10].

These results have been debated because of two main methodological limitations: (1) the composition of the abstainers' group to which moderate drinkers are generally compared and (2) the potential impact of confounding factors. First, the abstainers' group generally includes lifetime abstainers, former drinkers, and, in some studies, infrequent (and usually light) drinkers. However, since former and infrequent drinkers may stop or reduce their drinking because of health-related issues [17–22], health status possibly dictates drinking habits more than drinking habits affect health status. Thus, the potential benefits of moderate drinking may be artificially increased [23].

Secondly, critics have made the problem of potential confounding factors explicit; that is, the protective effect of moderate drinking could be partially explained by other determinants of health which are likely to be associated with a reduction in the risk of cardiovascular morbidity and mortality, such as education, socioeconomic and marital status, social network, psychological health, and other health-related behaviours (e.g., diet, nonsmoking habits, and exercising) [17, 18, 20, 21, 24–27]. For example, former drinkers (defined as those who did not drink in the past year) and long-term abstainers are more likely to be less educated, to be of a lower socioeconomic status than drinkers [18, 27–31], and to receive less social support [27, 32]. These characteristics are in turn related to higher mortality risks [33]. In addition, Naimi and colleagues [26] found that, among US adults from the general population, nondrinkers (defined as those who did not drink during the past 30 days) were more likely to have characteristics associated with increased cardiovascular mortality, such as reporting lower overall physical activity level, being overweight, having less access to health services, presenting comorbid health issues (diabetes and hypertension) and poorer general health status, and having a higher risk of developing cardiovascular diseases.

Subsequent studies have therefore either excluded former drinkers from the abstainer category or analyzed them separately. Yet lifetime abstainers remained at higher morbidity and mortality risk compared with moderate drinkers [23, 34]. They also controlled for confounding factors and have persisted in finding a significant relationship between moderate drinking and a lower risk for cardiovascular morbidity and mortality [8, 16]. Currently, the evidence indicates that moderate drinking is associated with better cardiovascular health, but the relationship may still be exacerbated by other determinants of health.

The objectives of the study are (1) to assess the relationship between older adults' drinking profiles and self-rated health, more specifically to compare moderate drinkers with other types of drinkers and nondrinkers, and (2) to assess whether and how this relationship is modified by other determinants of health including demographic, psychosocial, and health-related factors. Drinking profiles are expected to be significantly associated with self-rated health, with moderate drinkers perceiving themselves as healthier than other types of drinkers and nondrinkers. Differences between moderate drinkers and other types of drinkers and nondrinkers are expected to disappear when considering demographic, psychosocial, and health-related factors.

## 2. Materials and Methods

### 2.1. Participants and Procedure

This study is part of the international, collaborative GENACIS project (GENder, Alcohol, and Culture: an International Study) that emphasizes topics including alcohol consumption, beverage preferences, drinking consequences and contexts, reasons for drinking and abstaining, social networks, and psychological and physical health. In Canada, the survey was approved by the Centre for Addiction and Mental Health Research Ethics Board. It is based on a representative sample of 14,067 adults aged between 18 and 76 years from the 10 provinces [35]. A two-stage sampling method was used: households were first selected using random-digit dialing (RDD) and in instances where there were more than one adult in the household, the adult chosen as the survey respondent was the one whose birthday followed the survey date most closely. The survey was conducted between January 2004 and March 2005 with computer-assisted telephone interviewing (CATI). Respondents were interviewed in the Canadian official language of their choice (French or English). On average, interview duration was 25.64 minutes (SD = 7.46). The overall response rate was 52.8%. The present study included a subsample of older adults between the ages of 55 and 74 (*n* = 3,670) with a total of 1,494 men and 2,176 women. Only a small number of respondents were 75 or older, so it was decided not to include them in the analyses. Twenty-one male and 36 female respondents were excluded from the analyses because of discrepancies in their answers (i.e., interitem). The final subsample included 1,473 men and 2,140 women.

### 2.2. Measures

#### 2.2.1. Dependent Variable

Previous research has shown a strong association between self-rated health, a person's evaluation of his or her own general health, and other direct and indirect measures of health including health assessments performed by general physicians [36–40]. In this study, self-rated health is used as a proxy for actual health status. Self-rated health was assessed with the following question: “In general, compared to others your age, how has your physical health been in the last 12 months: excellent, very good, good, fair, or poor?” On a five-point scale, answers ranged from excellent (1) to poor (5).

#### 2.2.2. Independent Variables

*(1) Drinking Measures*. Among measures of alcohol consumption, current drinking was assessed with the following question: “Did you consume any alcohol beverages such as wine, beer, hard liquor, sherry, coolers, or any other beverages containing alcohol during the last 12 months?” To distinguish between lifetime abstainers and former drinkers, we used two specific questions: “Have you ever drunk alcohol?” (0 = no or 1 = yes) and “How old were you when you first drank alcohol, more than just a sip or a taste?”* Lifetime abstainers* are those who never drank alcohol and who never drank alcohol more than just a sip or a taste.* Former drinkers* are those who have drunk alcohol prior to last 12 months and who drank alcohol more than just a sip or a taste.

We used three alcohol consumption dimensions to build our drinking profiles typology: the usual frequency of drinking, the usual frequency of having five standard drinks or more per drinking day (binge drinking), and the usual quantity of drinking per drinking day. For the usual frequency of drinking and binge drinking, respondents were asked the following question: “During the last 12 months, how often did you usually have any kind of drink containing alcohol (how often did you usually have five drinks or more on a single day)?” Possible responses included the following: every day, 5-6 days a week, 3-4 days a week, 1-2 days a week, 1–3 days a month, less than once a month, or never. The first three categories were merged into one category (3 days a week or more) due to the small number of respondents drinking and binge drinking daily. For the usual quantity per drinking day, respondents were asked the following: “One drink means one 12 oz. of regular beer, 5 oz. of wine, 3 oz. of port, sherry or vermouth, one-and-a-half oz. of hard liquor or liquor, or one 12 oz. of cooler. In the past 12 months, on those days when you had any kind of beverage containing alcohol, how many drinks did you usually have?” Answers ranged from 0.8 (light beer) to 28 drinks per day in this subsample and were recoded into a three-category variable: (1) 1-2 (including 0.8) drinks per drinking day, (2) 3-4 drinks per drinking day, and (3) 5 drinks or more per drinking day.

To categorize drinkers, we used cross-observations of the three alcohol consumption dimensions. In addition to the lifetime abstainers and former drinkers, the final drinking profiles typology included three categories of drinkers: (1) frequent and moderate drinkers, (2) frequent and nonmoderate drinkers, and (3) infrequent drinkers.* Frequent and moderate drinkers* were those who usually drank once a week or more, who drank 1 or 2 standard drinks per drinking day, and who binge drank less than once a month (including never in the past 12 months). For the* frequent and nonmoderate drinkers*, two profiles were observed: (1) drinkers who drank once a week or more and who drank 3 standard drinks or more per drinking day (no matter the binge drinking frequency) and (2) drinkers who drank 1 or 2 standard drinks per drinking day and binge drank once a month or more. The* infrequent drinkers* are those who usually drink less than once a week. Thirty male and 19 female respondents were excluded because they presented missing values on one or more of the three alcohol consumption dimensions, which made their categorization impossible.

*(2) Demographic Measures*. Demographic measures include gender, age, marital status, level of education, and employment status. The income measure was excluded because the number of missing values was disproportionately high. Age was treated as a continuous variable ranging from 55 to 74. Marital status was divided into three categories: married/partnered, divorced/separated/widowed, and single/never married. Level of education was a four-category variable ranging from 1 (less than secondary education) to 4 (university degree). Employment status included the following three categories: working for pay, retired, and other. The “other” category referred to those going to school, caring for family, on disability, or unemployed.

*(3) Psychosocial Measures*. For the psychosocial measures, we assessed for the availability of at least one person with whom they felt comfortable talking to (a confidant) by asking the following: “(Apart from your spouse/partner/romantic partner), is there someone that you feel confident that you can talk to about an important personal problem?” Membership in any voluntary organizations or associations was assessed with the following: “Are you a member of any voluntary organizations or associations such as school groups, church social groups, community centers, ethnic associations or social, civic or fraternal clubs?” We evaluated respondents' perception of psychological health with the question: “How would you describe your overall emotional and mental health? In general, over the last 12 months, has it been excellent, very good, good, fair or poor?” (1 = excellent to 5 = poor).

*(4) Health-Related Measures*. Body mass index (BMI) was derived by dividing respondents' weight (kilograms) by height squared (meters). Weight and height were self-reported. We asked respondents the following: “Can you tell me how tall you are without shoes?” and “How much do you weight?” Scores were recoded into a four-category BMI variable based on the WHO classification [41]: (1) underweight (BMI less than 18.5), (2) normal weight (18.5 to 24.9), (3) overweight (25 to 29.9), and (4) obese (30 and above). Cigarette smoking status was assessed by asking two questions: “Have you ever been a cigarette smoker?” and “Have you smoked cigarettes during the past 12 months?” Cigarette smoking status was then recoded into a three-category variable: (0) never smoked, (1) smoked prior to the past 12 months, and (2) smoked within the past 12 months. To assess psychotropic drug use, we asked the following: (1) “In the past 12 months, did you take tranquilizers such as Valium or Ativan?”, (2) “In the past 12 months, did you take antidepressants such as Prozac, Paxil or Effexor?”, and (3) “In the past 12 months, did you take sleeping pills?” The first two questions were recoded into one variable to differentiate between those who used tranquilizers and/or antidepressants during the past year and those who did not.

### 2.3. Statistical Analyses

To meet the objectives of the study, we performed two sequential multiple linear regression analyses, one for men and one for women. The forced entry (enter) method was used since we had good theoretical reasons to include all the variables in each model simultaneously [42]. Analyses were performed using PASW (Predictive Analytics Software) Statistics 18. Correlations and cross-tabulations between variables were first verified to ensure the absence of multicollinearity.

First, to assess the relationship between drinking profiles (independent categorical variable) and self-rated health (dependent continuous variable), we entered the drinking profiles in the first regression model. To verify the hypotheses, we focused on the following: (a) the proportion of variance explained by the drinking profiles (*R* ^2^ value) and (b) the comparison between frequent/moderate drinkers (the reference category) with other types of drinkers and nondrinkers regarding self-rated health. This was done using unstandardized coefficients with confidence intervals (95% CIs).

Secondly, to determine if and how this relationship is modified by demographic, psychosocial, and health-related variables (independent variables), we entered these control variables in the second regression model (the adjusted model). Here, we emphasized the following: (a) the proportion of variance explained by the second model compared with the first one (*R* ^2^ model 2 minus *R* ^2^ model 1) and (b) the comparison between frequent/moderate drinkers and other types of drinkers and nondrinkers regarding self-rated health when adjusting for demographic, psychosocial, and health-related variables. Again, this was done using unstandardized coefficients with CIs.

## 3. Results

### 3.1. Drinking Variables

The upper part of Table 1 shows the sample's drinking characteristics by gender. Three quarters of men (73.9%) and 65.4% of women are current drinkers. The majority of men drink weekly (61.6%) whereas the majority of women drink monthly or less (60.1%). Binge drinking had occurred less than once a month or never during the past year among 77.8% of men and 94.7% of women. However, 13.1% of men report binge drinking 1–3 days a month and 9.2% on a weekly basis. For the quantity of drinking, 72.2% of men and 90.9% of women usually consume 1-2 drinks per drinking day, whereas 19.5% of men and 8.3% of women take 3-4 drinks per drinking day. Among men, 8.3% usually consume 5 drinks or more per drinking day whereas among women this proportion is very low (0.8%).

The distribution of respondents (rates) for each category of the drinking profiles typology is presented in the lower portion of Table 1. The lifetime abstainers' category includes 6% of men and 14.6% of women. About twenty percent of men and women are former drinkers. Infrequent drinkers have the highest rate among both men and women, respectively, 28.8% and 39.4%. A quarter of the men and 21.5% of women are frequent/moderate drinkers whereas 19.5% of men 4.2% of women are frequent/nonmoderate drinkers.

### 3.2. Demographic, Psychosocial, and Health-Related Variables

Table 2 shows the demographic, psychosocial, and health-related characteristics of the sample by gender. For marital status, the majority of men (72.5%) and women (57.2%) are married/partnered. Twenty percent of men and 35.4% of women are divorced/separated/widowed and around 7% of men and women are single/never married. Concerning education, about half the men and half the women have a secondary education or less whereas the other half have some post-secondary studies or a university degree. As for employment status, almost half the men are working for pay and the other half are retired. Among women, half are retired and the other half are working for pay (31.8%) and “other” (12.9%).

The majority of men (77%) and women (89.9%) have at least one person to confide in and around forty percent of men (39.4%) and women (43.8%) are members of voluntary organizations or associations. On average, men and women think they are in good to very good psychological and physical health (means ranging between 2 and 2.5). The BMI indicates that 0.6% of men and 2.2% of women are underweight, 29.1% and 43.7% are in the normal weight range, 51.2% and 35.1% are overweight, and 19% of men and women are obese. Cigarette smoking status shows that 29.5% of men and 45.7% of women never smoked, whereas 48.1% of men and 34.1% of women smoked prior to the last 12 months and about 20% of men and women smoked during the last year. Among men, 9.8% are tranquilizer/antidepressant users and 9.5% are sleeping pill users, and that holds true for, respectively, 15.6% and 12% of women.

### 3.3. Regression Analyses

Results of the regression analyses for men and women are presented in Tables 3 and 4, respectively. First models indicate that drinking profiles accounted for 3.3% (men: *R* ^2^ = 0.033) and 4.8% (women: *R* ^2^ = 0.048) of the variation in self-rated health. For both genders, compared with frequent/moderate drinkers, lifetime abstainers (men: 0.48, *p* < 0.01; women: 0.60, *p* < 0.001), former drinkers (men: 0.62, *p* < 0.001; women: 0.75, *p* < 0.001), infrequent drinkers (men: 0.30, *p* < 0.001; women: 0.37, *p* < 0.001), and frequent/nonmoderate drinkers (men: 0.22, *p* < 0.05; women: 0.34, *p* < 0.05) all had higher scores on the self-rated health measure, meaning all of these groups had a worse self-rated health (as a reminder: 1 = excellent to 5 = poor). Thus, frequent/moderate drinkers reported a significantly better self-rated health compared with all the other types of drinkers and nondrinkers.

The demographic, psychosocial, and health-related variables in the second (adjusted) models explained a large amount of the variation in self-rated health: 27.2% (*R* ^2^ = 0.305; 30.5% model 2 minus 3.3% model 1) among men and 28.1% (*R* ^2^ = 0.329; 32.9% minus 4.8%) among women. These second model variables explained a significantly greater proportion of the variance in self-rated health compared with drinking profiles in the first model. Lifetime abstainers (men: 0.29, *p* < 0.05; women: 0.33, *p* < 0.001) and former drinkers (men: 0.25, *p* < 0.01; women: 0.41, *p* < 0.001) were still likely to perceive their health as worse compared with frequent/moderate drinkers in the second model, but the difference between frequent/moderate drinkers and frequent/nonmoderate drinkers became not significant. The difference between frequent/moderate drinkers and infrequent drinkers also became not significant among men whereas it remained significant among women (0.20, *p* < 0.01).

## 4. Discussion

As expected, our results showed that frequent/moderate drinkers were likely to report a better self-rated health compared with other types of drinkers (frequent/nonmoderate and infrequent drinkers) and nondrinkers (lifetime abstainers and former drinkers). When the demographic, psychosocial, and health-related (control) variables were considered in the regression models, frequent/moderate drinkers were still more likely to report a better self-rated health compared with nondrinkers. However, differences between frequent/moderate drinkers and other types of drinkers disappeared, except for infrequent female drinkers who still reported worse self-rated health. Infrequent female drinkers may drink small quantities of alcohol that places them at the “nondrinking” level of lifetime abstainers and former drinkers.

These findings add to the very limited literature on self-rated health and alcohol among older adults. Our results are generally consistent with those from past cross-sectional studies among the adult population (as opposed to the* older* adults' population) from other alcohol drinking countries, which found a J-shaped relationship between alcohol consumption and suboptimal (poor) self-rated health [43–48] or an L-shaped relationship; that is, the higher the consumption of alcohol, the lower the presence of suboptimal health [49, 50]. Our results also corroborate those from a recent study that evaluated a sample of older adults specifically [51]. This cross-sectional and longitudinal study by Frisher and collaborators (2015) examined, among a representative sample of the England population aged 50 years and older, the association between drinking profiles (quantity and frequency) and self-rated health adjusting for gender, age, wealth, social class, education, household composition, smoking, and body mass index. Their results showed that the prevalence of poor self-rated health was highest among nondrinkers, that none of the drinking profiles were associated with poor self-rated health, and that drinking profiles did not predict self-rated health at ten-year follow-up. Also similarly to our results, they found that demographic variables were associated with self-rated health but did not substantially change the association between drinking profiles and self-rated health.

Our results suggest that drinking is related to a better self-rated health compared with nondrinking regardless of the drinking profile. A probable reason why we did not find any differences between frequent/moderate drinkers and other types of drinkers regarding self-rated health could be that the older adults from our sample did not report binge drinking frequently (i.e., frequency of having 5 drinks or more per drinking occasion) and were, in majority, very light to moderate drinkers. Another possible explanation could be that important lifestyle factors, which were not measured in our study, could have made a difference between drinking profiles regarding self-rated health, such as physical exercise and dietary habits. This would correspond to the well-known health risk factors (tobacco use, physical inactivity, unhealthy diet, and harmful use of alcohol) [52] and to the findings from a recent meta-analysis: compared with individuals with unhealthy lifestyle behaviors, such as abstaining from alcohol or excessive drinking, smoking, not exercising, having an unhealthy diet and being obese, individuals with at least four healthy lifestyle behaviors, including moderate drinking, not smoking, eating healthily (Mediterranean diet or regular eating of fruit/vegetables), exercising on a regular basis, and maintaining a normal weight, had a reduction in all-cause mortality risk by 66% [53]. Finally, we cannot exclude that our results might reflect the positive biological effect of alcohol on health. More data are needed to provide definitive evidence and clarify which explanation is the most plausible.

When distilling the vast literature on the subject, it makes sense that the benefits of drinking are conditional on other health practices and/or other life conditions. The relationship between alcohol and health requires taking into account the life-course drinking patterns and associated lifestyles factors [51].

This study has several limitations. First, the cross-sectional design makes causal interpretations impossible. The drinking profiles and other determinants of health were significantly associated with self-rated health, but the direction of this association is unknown. Second, the subsample used in this study was not a representative sample of older adults in Canada, thus limiting the ability to generalize our results. Third, we decided to exclude respondents aged 75 years and older from the analyses because there were too few of them in the sample. However, with the increasing longevity worldwide, more research is needed to better understand these older individuals' alcohol use. Fourth, self-reported data may have biases in recall and reporting. Memory errors, difficulties in the assessment of alcohol content, and the potential stigma associated with alcohol consumption may have led to an underestimation of drinking [54–56]. Fifth, although the self-rated health measure has been shown to be strongly correlated with actual health status [36–39], it does not assess physical health directly and empirically. Results have to be interpreted cautiously because they apply to the respondents' subjective perception of health. A final limitation of this study is its inability to classify former drinkers by their reasons for stopping drinking. This is due to the data collection: the survey was designed to ask only 50% of lifetime abstainers and former drinkers to provide reasons for abstaining. The number of cases in each category would have been too small to conduct analyses.

## 5. Conclusions

In this study among older adults, no evidence indicates that drinking alcohol is associated with poor self-rated health. Our results concur with comparable studies in other Western countries suggesting that alcohol drinking in old age, as is the case with most respondents in this study, may be a marker of good health. Nevertheless, the relationship is complex and other factors, both past and present in the life cycle, mediate this relationship. To avoid the oversimplification of the association between alcohol and health, the building of health-predicting models for mature and old adults will require integrated research that will examine drinking profiles and other determinants of health.

## Figures and Tables

**Table 1:** Drinking characteristics of men and women, 55–74 years of age (*n* = 3,613).

Drinking variables	Men	Women
	*n* = 1,473	*n* = 2,140
	(%)	(%)
Current drinkers	73.9	65.4
Frequency of drinking	*n* = 1,082	*n* = 1,389
Less than once a month	16.5	32.0
1–3 days per month	21.9	28.1
1-2 days per week	27.7	21.2
3+ days per week	33.9	18.6
Frequency of binge drinking	*n* = 1,072	*n* = 1,395
Never	55.8	84.2
Less than once a month	22.0	10.5
1–3 days per month	13.1	3.6
1-2 days per week	6.0	1.1
3+ days per week	3.2	0.6
Quantity of drinking	*n* = 1,063	*n* = 1,385
1-2 drinks per drinking day	72.2	90.9
3-4 drinks per drinking day	19.5	8.3
5+ drinks per drinking day	8.3	0.8
Drinking profiles	*n* = 1,443	*n* = 2,121
Lifetime abstainers	6.0	14.6
Former drinkers	20.7	20.3
Frequent/moderate drinkers	25.1	21.5
Frequent/nonmoderate drinkers	19.5	4.2
Infrequent drinkers	28.8	39.4

*Note*. The numbers (*n*) vary slightly because of missing responses.

**Table 2:** Demographic, psychosocial, and health-related characteristics (control variables) of men and women, 55–74 years of age (*n* = 3,613).

	Men	Women
	*n* = 1,473	*n* = 2,140
*Demographic variables*		
Marital status (%)	*n* = 1,469	*n* = 2,131
Married/partnered	72.5	57.2
Divorced/separated/widowed	19.9	35.4
Single/never married	7.6	7.4
Education (%)	*n* = 1,443	*n *= 2,094
Less than secondary	29.9	28.5
Secondary	23.2	28.0
Some post-secondary studies	20.4	24.9
University degree	26.4	18.6
Employment status (%)	*n* = 1,456	*n* = 2,101
Working for pay	45.9	31.8
Retired	47.9	55.3
Other	6.3	12.9
*Psychosocial variables*		
Having at least one confidant (%)	*n* = 1,441	*n* = 2,128
	77	89.9
Member of voluntary org./ass. (%)	*n* = 1,467	*n* = 2,134
	39.4	43.8
Self-rated psychological health^a^	*n* = 1,464	*n* = 2,129
(1 = excellent to 5 = poor)	M = 2.11 (SD = 0.96)	M = 2.23 (SD = 1.01)
*Health-related variables*		
Self-rated physical health^a^	*n* = 1,466	*n* = 2,135
(1 = excellent to 5 = poor)	M = 2.37 (SD = 1.19)	M = 2.46 (SD = 1.17)
Body mass index (BMI) (%)	*n* = 1,461	*n* = 2,044
Underweight	0.6	2.2
Normal weight	29.1	43.7
Overweight	51.2	35.1
Obese	19.1	19.0
Cigarette smoking status (%)	*n* = 1,473	*n* = 2,140
Never smoked	29.5	45.7
Smoked prior to last 12 months	48.1	34.1
Smoked within last 12 months	22.3	20.2
Tranquilizer/antidep. use (%)	*n* = 1,473	*n* = 2,140
	9.8	15.6
Sleeping pill use (%)	*n* = 1,473	*n* = 2,139
	9.5	12

^a^Ordinal variables, five categories, used as continuous variables in the analyses.

**Table 3:** Regression analysis of self-rated health among men, 55–74 years of age (*n* = 1,424).

Variables	*B*	95% confidence intervals	
		Lower	Upper
*Model 1*: *R* ^2^ = 0.033			
Drinking profiles			
Lifetime abst. versus freq./mod. drinkers	0.48^*∗∗*^	0.20	0.75
Former drinkers versus freq./mod. drinkers	0.62^*∗∗∗*^	0.44	0.80
Infreq. drinkers versus freq./mod. drinkers	0.30^*∗∗∗*^	0.13	0.46
Freq./nonmod. drinkers versus freq./mod. drinkers	0.22^*∗*^	0.04	0.40
*Model 2 (including control variables)*: *R* ^2^ = 0.305			
Drinking profiles			
Lifetime abst. versus freq./mod. drinkers	0.29^*∗*^	0.04	0.53
Former drinkers versus freq./mod. drinkers	0.25^*∗∗*^	0.09	0.41
Infreq. drinkers versus freq./mod. drinkers	0.10	−0.04	0.24
Freq./nonmod. drinkers versus freq./mod. drinkers	0.04	−0.12	0.20
Demographic variables			
Age	0.00	−0.01	0.01
Divorced/separated/widowed versus married/partnered	−0.04	−0.17	0.10
Single/never married versus married/partnered	−0.11	−0.31	0.10
Secondary versus less than secondary	−0.15^*∗*^	−0.30	−0.01
Some post-secondary versus less than secondary	−0.17^*∗*^	−0.32	−0.01
University degree versus less than secondary	−0.25^*∗∗*^	−0.40	−0.10
Retired versus working for pay	0.22^*∗∗*^	0.09	0.35
Other versus working for pay	0.78^*∗∗∗*^	0.54	1.01
Psychosocial variables			
Having at least one confidant	−0.06	−0.19	0.06
Member of voluntary org./ass.	−0.07	−0.18	0.04
Self-rated psychological health	0.48^*∗∗∗*^	0.43	0.54
(1 = excellent to 5 = poor)			
Health-related variables			
Underweight versus normal weight	0.40	−0.30	1.12
Overweight versus normal weight	0.14^*∗*^	0.02	0.26
Obese versus normal weight	0.52^*∗∗∗*^	0.37	0.68
Smoked prior to last 12 months versus never smoked	0.08	−0.05	0.20
Smoked within last 12 months versus never smoked	0.31^*∗∗∗*^	0.16	0.46
Tranquilizer/antidep. use	0.13	−0.06	0.32
Sleeping pill use	0.21^*∗*^	0.01	0.40

^*∗*^ *p* < 0.05, ^*∗∗*^ *p* < 0.01, ^*∗∗∗*^ *p* < 0.001.

*Note*.* B*: unstandardized coefficients. Higher scores mean worse self-rated health.

**Table 4:** Regression analysis of self-rated health among women, 55–74 years of age (*n* = 2,106).

Variables	*B*	95% confidence intervals	
		Lower	Upper
*Model 1*: *R* ^2^ = 0.048			
Drinking profiles			
Lifetime abst. versus freq./mod. drinkers	0.60^*∗∗∗*^	0.43	0.76
Former drinkers versus freq./mod. drinkers	0.75^*∗∗∗*^	0.60	0.90
Infreq. drinkers versus freq./mod. drinkers	0.37^*∗∗∗*^	0.24	0.50
Freq./nonmod. drinkers versus freq./mod. drinkers	0.34^*∗*^	0.08	0.60
*Model 2 (including control variables)*: *R* ^2^ = 0.329			
Drinking profiles			
Lifetime abst. versus freq./mod. drinkers	0.33^*∗∗∗*^	0.18	0.48
Former drinkers versus freq./mod. drinkers	0.41^*∗∗∗*^	0.28	0.54
Infreq. drinkers versus freq./mod. drinkers	0.20^*∗∗*^	0.09	0.31
Freq./nonmod. drinkers versus freq./mod. drinkers	0.09	−0.13	0.32
Demographic variables			
Age	0.00	−0.01	0.01
Divorced/separated/widowed versus married/partnered	0.01	−0.08	0.10
Single/never married versus married/partnered	0.08	−0.08	0.24
Secondary versus less than secondary	−0.12^*∗*^	−0.23	−0.01
Some post-secondary versus less than secondary	−0.07	−0.19	0.05
University degree versus less than secondary	−0.17^*∗*^	−0.31	−0.04
Retired versus working for pay	0.16^*∗∗*^	0.05	0.27
Other versus working for pay	0.48^*∗∗∗*^	0.34	0.62
Psychosocial variables			
Having at least one confidant	−0.09	−0.23	0.05
Member of voluntary org./ass.	−0.03	−0.11	0.06
Self-rated psychological health	0.45^*∗∗∗*^	0.41	0.50
(1 = excellent to 5 = poor)			
Health-related variables			
Underweight versus normal weight	0.10	−0.20	0.41
Overweight versus normal weight	0.12^*∗∗*^	0.03	0.22
Obese versus normal weight	0.42^*∗∗∗*^	0.30	0.54
Smoked prior to last 12 months versus never smoked	0.08	−0.01	0.18
Smoked within last 12 months versus never smoked	0.17^*∗∗*^	0.05	0.28
Tranquilizer/antidep. use	0.32^*∗∗∗*^	0.19	0.44
Sleeping pill use	0.26^*∗∗∗*^	0.12	0.39

^*∗*^ *p* < 0.05, ^*∗∗*^ *p* < 0.01, ^*∗∗∗*^ *p* < 0.001.

*Note. B*: unstandardized coefficients. Higher scores mean worse self-rated health.
